# DFS: A Feature–Sample Collaborative Optimization Framework for Machine Learning-Based Forest Aboveground Biomass Estimation Using Multi-Source Remote Sensing

**DOI:** 10.3390/plants15152387

**Published:** 2026-08-04

**Authors:** Yi Zhu, Zilin Ye, Peisong Yang, Ziqing Ye, Guoxiong Zhou

**Affiliations:** 1 College of Computer and Mathematics, Central South University of Forestry and Technology, Changsha 410004, China; 20234345@csuft.edu.cn; 2 College of Forestry, Central South University of Forestry and Technology, Changsha 410004, China; 20230100006@csuft.edu.cn (Z.Y.); 20210100004@csuft.edu.cn (P.Y.); 3 College of Electronic Information and Physics, Central South University of Forestry and Technology, Changsha 410004, China

**Keywords:** forest carbon monitoring, satellite remote sensing, feature selection, active learning, biomass mapping, ecological modeling

## Abstract

High-precision estimation of forest aboveground biomass (AGB) is crucial for global carbon cycle monitoring and sustainable forest management. However, existing machine learning-based approaches often suffer from high-dimensional feature redundancy, uneven spatial distribution of training samples, and inefficient hyperparameter optimization, which collectively limit estimation accuracy and computational efficiency. To address these issues, this study proposes a synergistic feature-sample optimization framework (DFS) for high-precision forest AGB estimation. First, with the involvement of forestry experts, we constructed the Hunan and Hubei datasets covering typical subtropical forest types through multi-source remote sensing and ground plot sampling. Second, we propose the Dual-Criteria Adaptive Feature Selection (DCAFS) method, integrating ReliefF and mutual information criteria to adaptively select key features highly correlated with AGB, eliminating spectral redundancy while preserving biomass-sensitive information. Next, we introduce a Bidirectional Active Learning Sample Optimization mechanism, called BALSO, and in its forward step, plots with high uncertainty and representativeness are given priority, so samples with high AGB variability can be captured effectively; in the backward step, spatially redundant samples and feature-redundant samples are removed through density peak clustering, and by doing this, sample selection and spatial distribution are optimized at the same time, so plot balance gets improved. Finally, the framework brings in a parameter tuning structure based on Dream Optimization Algorithm, namely DOA, and through staged exploration together with local fine-tuning, DOA makes model hyperparameters and AGB data distribution characteristics align in an adaptive manner, which helps improve convergence efficiency and estimation stability. Input variables comprise Landsat 8 OLI spectral bands, GLCM texture features, vegetation indices, and Sentinel-1/2 data. On the Hunan dataset, the framework achieved an $R^{2}$ of 0.83 and an RMSE of 25.6 Mg·ha^−1^; on the Hubei dataset, it achieved an $R^{2}$ of 0.86 and an RMSE of 26.8 Mg·ha^−1^. The framework was further validated on an independent public dataset from Inner Mongolia. These results demonstrate that the DFS framework provides an effective and feasible approach for regional-scale forest AGB estimation and carbon monitoring.

## 1. Introduction

Forest aboveground biomass (AGB) is a fundamental component of forest carbon storage. It plays a central role in global carbon cycle assessment, and sustainable forest management [1,2]. AGB does not represent a single forest attribute. Instead, it reflects the cumulative outcome of tree growth, stand structure, and ecosystem functioning [3,4]. Remote sensing estimation of AGB essentially translates observable biophysical signals into quantitative biomass information [5]. Therefore, improving the accuracy and robustness of AGB estimation is important for forest resource assessment. It is also critical for understanding ecosystem dynamics and supporting carbon-neutrality strategies at regional and global scales [6].

Recent advances in remote sensing have expanded the capability of observing forest biophysical properties. Optical observations mainly characterize vegetation biochemical properties [7]. SAR is more sensitive to canopy structure and water content [8,9]. LiDAR directly captures three-dimensional forest architecture [10,11]. Integrating multi-source remote sensing data has become an effective strategy for improving AGB estimation [12,13]. Machine learning techniques have been widely adopted to model nonlinear relationships between remotely sensed observations and forest biomass. They can capture complex interactions among heterogeneous variables [14]. Recent studies have demonstrated that combining multi-source observations with machine learning algorithms enhances AGB estimation. This approach works well across diverse forest ecosystems [15,16,17].

Despite remarkable progress in combining multi-source remote sensing with machine learning, the reliability of AGB estimation is still limited by the complexity of modeling workflows. A core bottleneck lies in the widespread redundancy of high-dimensional remote sensing variables: massive features extracted from optical, radar, topographic and texture data characterize analogous forest biophysical properties and suffer severe inter-feature correlation. Accordingly, feature selection becomes an indispensable step in this workflow [18]. Existing approaches fall into three categories: filter, wrapper and embedded methods. Filter techniques assess feature relevance directly via statistical metrics with high computational efficiency [19]; wrapper algorithms iteratively screen informative feature subsets driven by predictive performance yet incur heavy computational costs [20]; embedded schemes execute feature selection simultaneously during model training to strike a balance between efficiency and estimation accuracy [21]. Explainable machine learning tools such as SHAP further improve their adaptability to high-dimensional datasets [22]. However, all these strategies essentially treat observational samples as independent covariates and neglect the spatial structural patterns of AGB.

At the same time, the representativeness of training samples has become another critical factor influencing the generalization capability of AGB estimation models. Forest ecosystems are characterized by pronounced spatial heterogeneity arising from differences in species composition, stand age, topography, climate, and management history. Consequently, samples collected from limited regions or forest types often fail to capture the full ecological variability of the study area. Active learning and sample selection strategies have therefore been introduced to improve labeling efficiency by preferentially selecting informative or uncertain samples [23,24,25,26]. Although these methods reduce annotation costs and improve learning efficiency, most existing strategies determine sample importance mainly according to prediction uncertainty or statistical diversity, while paying limited attention to the interaction between sample representativeness and feature selection. Consequently, the ecological information preserved through optimized samples may not be fully exploited when redundant variables remain in the feature space.

Similarly, hyperparameter optimization has been widely employed to improve model performance through grid search, random search, Bayesian optimization, particle swarm optimization, and other intelligent optimization algorithms [27]. These approaches are effective in identifying parameter configurations that enhance predictive accuracy under a given modeling setting. However, the optimal hyperparameter configuration is inherently dependent on both the selected feature subset and the distribution of the training samples. Changes in either component inevitably alter the learning characteristics of the model, implying that parameter optimization performed independently may not remain optimal after modifications to features or samples. Therefore, although feature selection, sample optimization, and hyperparameter tuning have each been extensively investigated, they are commonly treated as separate stages of the modeling pipeline, and the interactions among them remain insufficiently explored in forest AGB estimation.

The above studies have substantially advanced AGB estimation by improving different stages of the modeling pipeline. In fact, feature selection, sample optimization, and hyperparameter tuning are inherently interconnected during model construction. Feature selection determines which biophysical information is retained for model learning, whereas the representativeness of training samples directly shapes the evaluated importance of candidate features. In turn, the selected features and training samples jointly govern the optimal model configuration and learning patterns. Nevertheless, existing studies mostly treat these three modules as separate tasks and pay limited attention to their synergistic effects within an integrated modeling framework.

To solve the problem of high-dimensional feature redundancy recently, metaheuristic optimisation algorithms are widely used due to their global search capability and parameter adaptability in high-dimensional feature selection, especially genetic algorithms and particle swarm optimisation [19,28]. Specifically, Chen et al. propose a “problem-aware particle swarm optimisation” [29] approach that dynamically adjusts search parameters by integrating problem structure information, which demonstrates better adaptability to high-dimensional classification and regression problems compared to traditional methods. Additionally, regularized regression approaches such as the optimized LASSO method proposed by Wang et al. [30] have shown promise in improving feature selection for AGB estimation. However, these algorithms have not yet been validated in the field of AGB estimation, and their interpretability under multi-source, multi-scale, heterogeneous forest landscape conditions remain to be further explored [22].

To address the issues of sample scarcity and uneven distribution, active learning has been successfully applied to remote sensing inversion tasks [23,24]. Persello et al. [25] proposed cost-sensitive active learning for field survey optimisation, and Shu et al. [26] specifically addressed sample optimisation for regional-scale forest AGB estimation. However, existing AGB estimation methods predominantly employ forward selection strategies—selecting high-uncertainty or high-representativeness samples from unlabeled pools—while neglecting redundancy accumulation in labeled sets [31]. This unidirectional mechanism introduces distribution bias [32]. Although bidirectional active learning enables simultaneous sample addition and deletion, its application to forest AGB estimation remains limited: it fails to couple environmental distributions with feature space distributions, and sample compression compromises environmental representativeness, thereby degrading model generalization [33].

In terms of model parameter optimization, recent studies have introduced cognition-inspired mechanisms to boost the efficiency of adaptive optimization for algorithms within complex search spaces. Mind Evolutionary Algorithm (MEA) simulates the convergence and divergence processes in human thinking, balancing exploration and development through intra-population cooperation and inter-population competition [34]; Memetic Algorithm combines global search with local heuristic rules, drawing on the imitation and learning mechanisms in cultural transmission [35]. Additionally, Brain Storm Optimization (BSO) is inspired by the collision of ideas and clustering in collective decision-making, generating new solutions through clustering and selecting high-quality individuals to guide the search direction [34]. Although these methods show potential in some optimization problems, they still have limitations in geospatial data analysis: most algorithms have sensitive parameter settings, insufficient adaptive optimization capabilities for high-dimensional heterogeneous data, and are easily affected by data distribution [27].

To address the above challenges, the main contributions of this article are as follows:

1. We constructed a multi-regional forest aboveground biomass dataset covering typical subtropical forests in Hunan and Hubei, China. This dataset integrates multi-source remote sensing data with field survey plots, and all samples were rigorously annotated by forestry experts to provide a benchmark dataset for method validation.
2. We propose the Dual-Criteria Adaptive Feature Selection method, DCAFS. Inspired by the problem-aware optimization concept, DCAFS is built on the particle swarm optimization framework and constructs a dynamic weighting strategy that combines ReliefF and mutual information. This approach embeds feature importance assessment into the parameter adjustment process, where the inertia weight and acceleration coefficients are adaptively tuned based on each feature’s discriminative capability for AGB. DCAFS is aimed at screening key features from multi-source remote sensing data, with the goal of reducing spectral redundancy while preserving biomass-sensitive information.
3. We design the Bidirectional Active Learning Sample Optimization mechanism, BALSO. In the forward selection stage, BALSO gives priority to samples with higher information content by integrating uncertainty sampling with representativeness metrics. In the backward elimination stage, an improved density peak clustering algorithm is employed to identify and remove redundant samples. This bidirectional strategy is intended to mitigate sample imbalance and environmental distribution bias.
4. We introduce an adaptive collaborative search strategy. This structure puts staged exploration and local fine-tuning together, so that the parameter space can be navigated in an efficient way and convergence behaviour can be improved across different sample sizes and feature dimensions.

## 2. Materials and Methods

### 2.1. Study Area

#### 2.1.1. Study Areas for Self-Constructed Datasets

**Figure 1:**
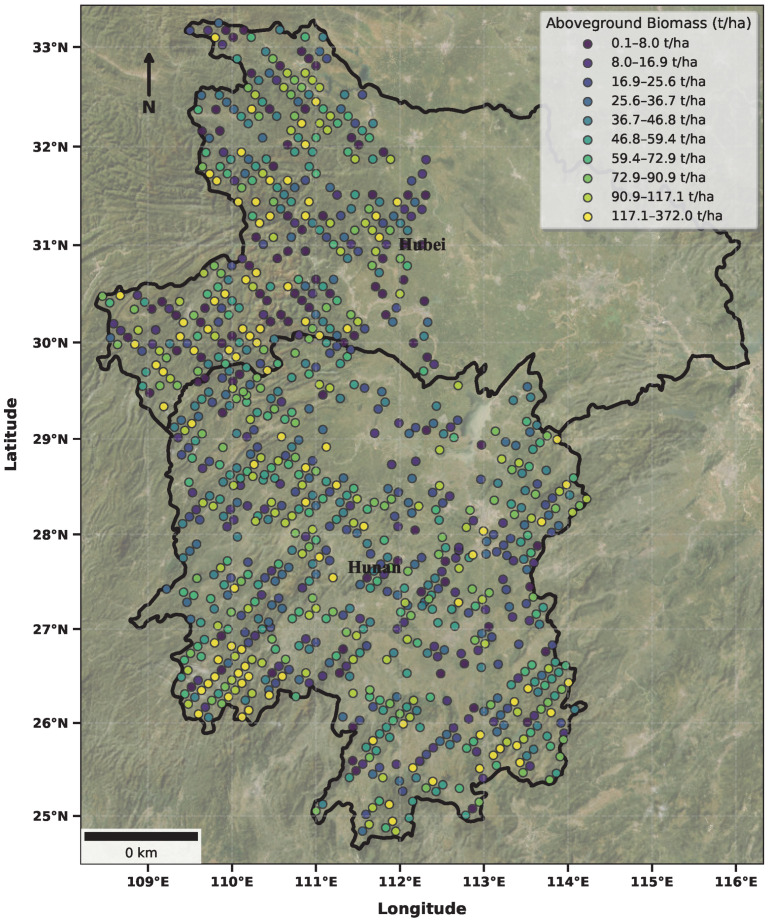
Study area map of Hunan and Hubei provinces showing the spatial distribution of 937 forest continuous inventory field sample plots overlaid on a topographic hillshade base map, with plot colors indicating measured AGB (t·ha^−1^).

This self-built dataset was collected from typical subtropical forests in Central China, and the study area covers parts of Hunan and Hubei provinces (Figure 1). The geographical range is approximately 108°–114° E and 26°–31° N, with a total area of about 100,000 km^2^. The climate here is subtropical monsoon, the mean annual temperature is 14–18 °C, and the annual precipitation ranges from 1000 to 1600 mm. The terrain is mainly hilly and mountainous, with elevations between 100 and 2000 m. The vegetation types are mainly evergreen broadleaf forests, evergreen-deciduous broadleaf mixed forests, and conifer-broadleaf mixed forests. The dominant tree species include Chinese fir (*Cunninghamia lanceolata*), Masson’s pine (*Pinus massoniana*), camphor tree (*Cinnamomum camphora*), and Chinese chestnut oak (*Cyclobalanopsis glauca*) [12].

#### 2.1.2. Study Area for Public Dataset

In order to test whether the proposed method can work in different places, this study also used the public dataset from Wangyedian Experimental Forest Farm in Inner Mongolia, and this dataset was provided by Xu et al. [20]. The study area is located in Chifeng City, Inner Mongolia Autonomous Region, its geographic coordinates are from 118°09′ to 118°30′ E and from 41°21′ to 41°39′ N, and the total area is about 25,958 hectares. This place has a mid-temperate continental monsoon climate, the average annual sunshine duration there reaches 2913.3 h. The terrain is mainly hilly, and the elevation ranges from 800 m to 1890 m. The forest cover in this area is about 93%, and the dominant tree species are larch (*Larix principis-rupprechtii* and *Larix olgensis*) as well as Chinese pine (*Pinus tabuliformis*).

### 2.2. Data Sources and Preprocessing

#### 2.2.1. Self-Constructed Dataset

We built this dataset ourselves, and it combines geospatial data from multiple sources with field survey data, the study areas are some typical subtropical forest areas in Hunan and Hubei provinces of China.

Remote sensing data were acquired from publicly available Landsat 8 OLI/TIRS Collection 2 Level-2 Surface Reflectance (SR) images via the Google Earth Engine (GEE) platform(Google LLC, Mountain View, CA, USA). These SR products have undergone radiometric calibration and atmospheric correction by the U.S. Geological Survey (USGS). Images from the 2021 growing season covering the study area were collected, and scenes with cloud cover below 10% were selected. In ENVI 5.6 software(L3Harris Geospatial, Melbourne, FL, USA), the downloaded scenes were mosaicked and clipped to the study area boundaries. After that, we extracted reflectance values from multiple spectral bands. Then we computed texture features at multiple scales using the Gray-Level Co-occurrence Matrix (GLCM) in ENVI, the window sizes we used were 5 × 5, 7 × 7, and 9 × 9, so that we could get structural information at different spatial scales. We also calculated ten vegetation indices that are commonly used, such as NDVI, EVI, and SAVI, and these were based on the red, near-infrared, and shortwave infrared bands (Table 1). These indices enhance the characterization of forest canopy biophysical properties.

Ground truth data came from forest continuous inventory plots, and these plots were provided by the Central South Forestry Survey and Planning Institute of the National Forestry and Grassland Administration. The original plot data were unified to a common coordinate system and registered in ArcGIS 10.2(Esri, Redlands, CA, USA). This step ensured alignment with the remote sensing imagery. After rigorous quality screening and outlier removal, 937 valid plots remained. Of these, 625 were in Hunan Province and 312 were in Hubei Province. Each plot measured 30 m × 30 m and covered the major forest types in the study area. Key stand parameters were recorded for each plot. These included diameter at breast height (DBH), tree height, stand density, dominant tree species, and age class. Plot-level AGB was calculated with species-specific allometric equations.

Finally, the spectral features, textural features, and vegetation indices extracted from the remote sensing data were spatially linked to the plot locations using the ArcGIS platform. Then we exported the resulting dataset as a feature table, so that we could use it for the modeling analysis later. Before putting the data into the model, we normalized all numerical features with Z-score standardization, this step was done to remove the influence caused by different dimensional units.

#### 2.2.2. Public Dataset

To test whether the proposed method works on other data, this study used a public dataset from the Wangyedian Experimental Forest Farm in Inner Mongolia, and this dataset was provided by Xu et al. [20]. It combines remote sensing data from different sources with ground measurements.

The remote sensing data came from Sentinel-1A (Level-1 GRD) and Sentinel-2A (Level-1C), and these images were taken in September 2017, which can be downloaded from the Copernicus Data Space Ecosystem (https://dataspace.copernicus.eu/, accessed on 9 July 2025). For the Sentinel-1A data, we did radiometric calibration, Lee sigma filtering (the window was 7 × 7), and range-Doppler terrain correction. The Sentinel-2A data were corrected for radiometric, geometric, and atmospheric issues. All data were resampled to 25 m spatial resolution, and this was done in a uniform way.

Ground truth data were obtained from 81 sample plots located within the Wangyedian Experimental Forest Farm, including plot locations, tree DBH, tree height, individual tree volume, and total growing stock volume (GSV) per plot. Due to privacy and confidentiality restrictions, these ground-based measurements have not been made publicly available; however, authorization for their use in this study was granted by the corresponding author. Plot-level AGB was calculated using species-specific allometric equations, providing a reliable benchmark for validation.

The dataset and associated code have been made publicly available by the original authors on the Zenodo platform (DOI: 10.5281/zenodo.5641318), enabling other researchers to validate and further refine the data processing methodologies.

### 2.3. Overall DFS Framework

This study proposes the DFS framework (Feature–Sample Collaborative Optimization Framework). DCAFS dynamically adjusts particle swarm parameters using ReliefF and mutual information dual criteria, effectively reducing feature dimensions while preserving key biophysical features, thereby providing high-quality input for subsequent estimation. BALSO optimizes sample distribution and addresses spatial redundancy issues through a bidirectional iterative mechanism combining forward uncertainty-representativeness sampling and backward redundancy elimination. DOA intelligently adjusts model hyperparameters based on a dream memory mechanism, ensuring optimal performance across different scenarios. This integrated approach enables DFS to achieve high-precision estimation at significantly lower computational costs compared to deep learning methods.

**Figure 2:**
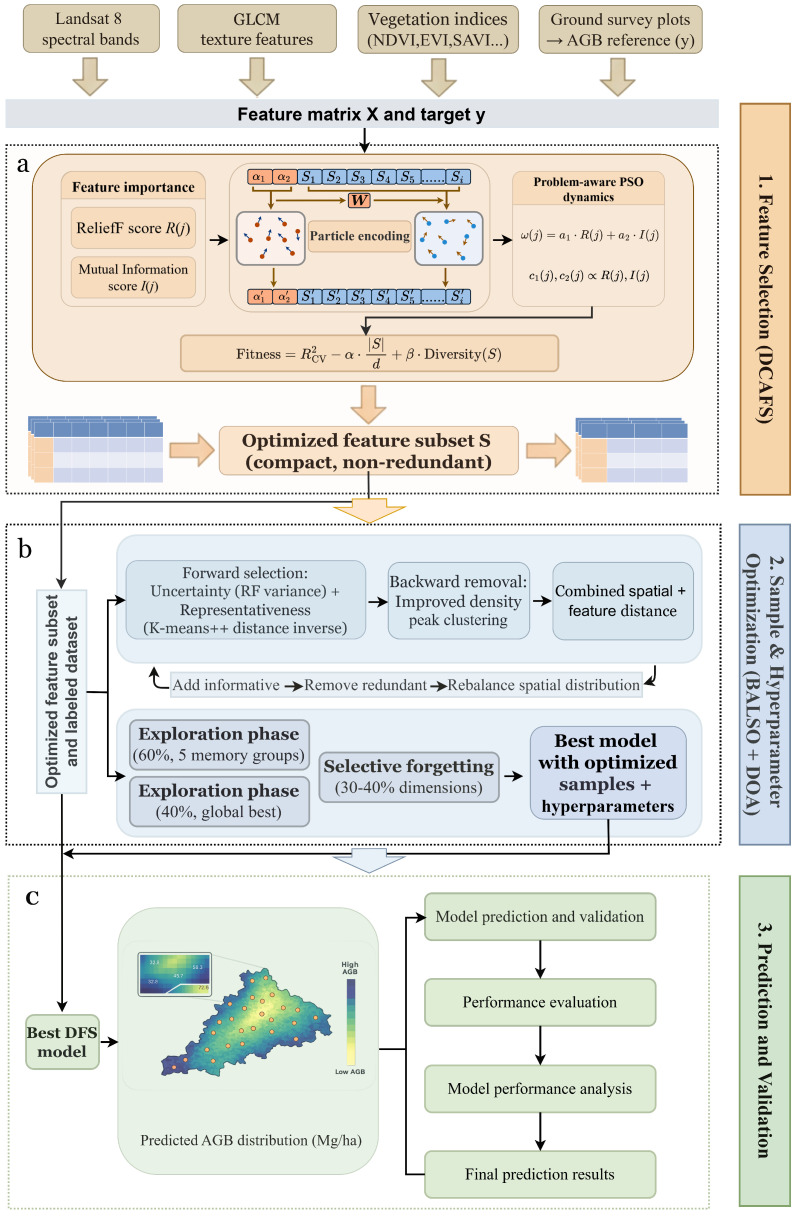
The DFS framework integrates three core modules to achieve high-precision forest AGB estimation through feature-sample-parameter collaborative optimization. (**a**) DCAFS module integrates ReliefF and mutual information dual criteria into a problem-aware PSO framework to adaptively select key features and eliminate spectral redundancy. (**b**) BALSO module employs bidirectional active learning with forward uncertainty-representativeness sampling and backward redundancy elimination to optimize sample distribution in feature space, while DOA module applies dream optimization algorithm with memory-forgetting mechanism for hyperparameter tuning. (**c**) Prediction and validation generates AGB distribution maps with quantitative metrics demonstrating improved accuracy across study areas.

Specifically, the DFS framework includes the following four core modules:

1. The DCAFS module (Figure 2a) adopts a problem-aware particle swarm optimization algorithm to dynamically adjust PSO parameters through the dual criterion of ReliefF and mutual information, and adaptively screen the optimal feature subset from the original feature set, eliminating feature redundancy while retaining key biophysical information.
2. The BALSO module (Figure 2b) is based on the two-way active learning framework, and intelligently optimizes the distribution of training samples through the two-way iterative mechanism of forward uncertainty-representative sampling and backward redundancy elimination, and solves the problems of sample imbalance and spatial redundancy.
3. The DOA module (Figure 2b) introduces a dream optimization algorithm to simulate the memory-forgetting mechanism of human dreams, and intelligently tunes the hyperparameter combination of the machine learning model through the grouped memory strategy and selective forgetting mechanism.
4. Model training and validation: Train the machine learning model using the optimized feature-sample-parameter combination and conduct comprehensive performance evaluation.

### 2.4. DCAFS

In the initialization phase, DCAFS first evaluates how important each feature is by using ReliefF and MI, and then normalization is done. ReliefF captures local feature importance by looking at discriminative ability within neighborhoods, and MI quantifies the global statistical dependence between features and the AGB target variable. These two criteria are put together, so the algorithm can balance local discriminability and global relevance, and this gives multi-perspective feature importance priors for subsequent parameter adjustments.

The core innovation of DCAFS is its problem-aware parameter adjustment mechanism. DCAFS defines ω as a dynamic variable that is based on feature importance:.

$$\omega \left(j\right)=a_{1}\cdot R\left(j\right)+a_{2}\cdot I\left(j\right)$$

where R(j) and I(j) are the normalized ReliefF and MI scores for feature *j*, respectively, and $a_{1},a_{2}$ are weighting parameters controlling the relative contributions of each criterion.

To enable joint optimization of the feature subset and the criterion weighting, each particle in DCAFS is encoded as a hybrid vector that simultaneously represents both the feature selection mask and the adaptive weighting parameters:

$$X_{i}=\left[x_{i,1},x_{i,2},\ldots ,x_{i,d},a_{i,1},a_{i,2}\right]$$

where $x_{i,j}\in \left\{0,1\right\}$ (j=1,…,d) is the binary selection indicator for feature *j*, and $a_{i,1}$, $a_{i,2}\in \left[0,1\right]$ are continuous weighting parameters subject to the constraint $a_{i,1}+a_{i,2}=1$. This hybrid encoding enables the PSO to optimize the feature subset and the criterion weighting in a single search process.

During initialization, the feature selection variables $x_{i,j}$ are randomly set to 0 or 1 with equal probability. The weighting parameters are initialized by sampling $a_{i,1}^{\prime },a_{i,2}^{\prime }∼U\left(0,1\right)$ independently and then normalizing:

$$a_{i,1}=\frac{a_{i,1}^{\prime }}{a_{i,1}^{\prime }+a_{i,2}^{\prime }},\;a_{i,2}=\frac{a_{i,2}^{\prime }}{a_{i,1}^{\prime }+a_{i,2}^{\prime }}$$

This ensures $a_{i,1}+a_{i,2}=1$ while maintaining uniform randomness in the relative weighting.

Regarding acceleration coefficients, DCAFS initializes them from the statistical properties of ReliefF and MI. Specifically, the feature-specific individual learning factor $c_{1}\left(j\right)$ and social learning factor $c_{2}\left(j\right)$ are obtained from the mean and standard deviation of ReliefF and MI scores for each feature *j*, respectively, and they are then mapped to the predefined interval $\left[c_{\min},c_{\max}\right]$ through min–max normalization.

DCAFS uses the following composite index as its optimization objective for feature selection:

$$\mathrm{Fitness}=R_{\mathrm{CV}}^{2}-\alpha \cdot \frac{\left|S\right|}{d}+\beta \cdot \mathrm{Diversity}\left(S\right)$$

where $R_{\mathrm{CV}}^{2}$ is the regression accuracy from 5-fold cross-validation, |S|/d is a penalty term for the feature selection rate, and Diversity(S) is a reward term that quantifies the complementarity among selected features. Specifically, Diversity(S) is defined as one minus the mean absolute Pearson correlation coefficient among the selected features:

$$\mathrm{Diversity}\left(S\right)=1-\frac{2}{\left|S|(|S|-1\right)}\sum_{1\leq i<j\leq |S|}\left|\rho_{ij}\right|$$

where $\rho_{ij}$ denotes the Pearson correlation coefficient between the *i*-th and *j*-th selected features, and |·| denotes the absolute value. This ensures that both positive and negative correlations are treated as redundancy, rewarding feature subsets with complementary information. The coefficients α and β are empirical balancing parameters that regulate the trade-off among prediction accuracy, feature compactness, and feature diversity in the fitness function. Following preliminary exploratory experiments, they were fixed to 0.3 and 0.2, respectively, and kept unchanged for all datasets and comparative experiments to ensure methodological consistency.

In order to enhance search diversity and avoid premature convergence, DCAFS introduces a random perturbation term into the particle velocity update formula for the binary feature selection variables:

$$\begin{array}{cc} V_{i,j}^{t+1}= & \;\omega \left(j\right)\cdot V_{i,j}^{t}+c_{1}\left(j\right)\cdot r_{1}\cdot \left(Pbest_{i,j}-x_{i,j}^{t}\right)+c_{2}\left(j\right)\cdot r_{2}\cdot \left(Gbest_{j}-x_{i,j}^{t}\right)-r_{3}\cdot \left(T_{j}-x_{i,j}^{t}\right) \end{array}$$

where *T* is an independently generated binary vector with each component satisfying $P\left(T_{j}=0\right)=P\left(T_{j}=1\right)=0.5$, and $r_{3}$ is independently sampled from a continuous uniform distribution over the interval [0,0.5], which controls the magnitude of the stochastic perturbation during particle position updating.

For the continuous weighting parameters $a_{i,1}$ and $a_{i,2}$, the standard continuous PSO update is applied:

$$\begin{array}{cc} V_{i,a_{k}}^{t+1} & =\omega_{\mathrm{global}}\cdot V_{i,a_{k}}^{t}+c_{1}\cdot r_{1}\cdot \left(Pbest_{i,a_{k}}-a_{i,k}^{t}\right)+c_{2}\cdot r_{2}\cdot \left(Gbest_{a_{k}}-a_{i,k}^{t}\right),\\ a_{i,k}^{t+1} & =a_{i,k}^{t}+V_{i,a_{k}}^{t+1}, \end{array}$$

where k∈{1,2} denotes the weighting parameter index, $\omega_{\mathrm{global}}=0.9-0.5\times \left(t/T_{\max}\right)$ is a linearly decreasing global inertia weight, and $c_{1},c_{2}$ are the standard acceleration coefficients set to 2.0. After each update, the parameters are normalized to preserve the unit-sum constraint:

$$a_{i,1}^{t+1}=\frac{\max(0,a_{i,1}^{t+1})}{\max\left(0,a_{i,1}^{t+1}\right)+\max\left(0,a_{i,2}^{t+1}\right)},\;a_{i,2}^{t+1}=\frac{\max(0,a_{i,2}^{t+1})}{\max\left(0,a_{i,1}^{t+1}\right)+\max\left(0,a_{i,2}^{t+1}\right)}$$

Normalization with max(0,·) bounds $a_{1}$ and $a_{2}$ to [0,1] while preserving their interpretability as relative contribution ratios.

The fitness of each particle is evaluated using the selected feature subset (determined by $x_{i,j}$) with the dynamic weighting parameters $a_{i,1}$ and $a_{i,2}$ incorporated into the inertia weight calculation. Both the feature mask and the weighting parameters are therefore subject to PSO optimization, with the best-performing combination emerging through the evolutionary search.

DCAFS puts ReliefF and MI together into a dual-criteria framework, and this enables dynamic and adaptive tuning of PSO parameters, so a fully problem-aware feature selection method is formed.

### 2.5. BALSO

The main of BALSO is its synergistic bidirectional optimization. In the forward selection phase, the algorithm gives priority to those samples that are most informative, and these samples are taken from an unlabeled pool U and then added to the training set L. To do this, both the uncertainty and representativeness of each sample need to be evaluated in a comprehensive way. Uncertainty is measured by the variance in predictions from a random forest model that was trained temporarily, and this variance represents the prediction entropy for an unlabeled sample $x_{i}$:

$$\mathrm{Uncertainty}\left(x_{i}\right)={\mathrm{Var}}_{\mathrm{trees}}\left[f\left(x_{i}\right)\right]$$

where $f(x_{i})$ denotes the set of predictions for sample $x_{i}$ from all decision trees in the random forest. Representativeness is assessed by applying K-means++ clustering to all samples in the feature space, and then the inverse of the Euclidean distance from a sample to its corresponding cluster center $c_{k}$ is computed. A smaller distance indicates that the representativeness is stronger:

$$\mathrm{Representativeness}\left(x_{i}\right)=\frac{1}{∥x_{i}-c_{k}{∥}_{2}}$$

These two metrics are then combined through a linear weighting, and they form a comprehensive priority score. This score gives preference to samples that exhibit “high uncertainty and high representativeness”:

$${\mathrm{Score}}_{\mathrm{forward}}\left(x_{i}\right)=0.6\times \mathrm{Uncertainty}\left(x_{i}\right)+0.4\times \mathrm{Representativeness}\left(x_{i}\right)$$

Uncertainty and representativeness were weighted 0.6 and 0.4, respectively, based on preliminary experiments; uncertainty was prioritized for information gain, while representativeness prevented isolated selections.

In the backward elimination phase, BALSO identifies redundant samples from the current training set L and removes them, so the information density of L can be enhanced. This phase uses an improved density peak clustering algorithm, and it performs a fine-grained partitioning of L. First, a comprehensive distance $d_{\mathrm{comprehensive}}$ is calculated, and it integrates both spatial proximity and feature similarity:

$$d_{\mathrm{comprehensive}}\left(i,j\right)=0.3\times d_{\mathrm{spatial}}\left(i,j\right)+0.7\times d_{\mathrm{feature}}\left(i,j\right)$$

where $d_{\mathrm{spatial}}$ and $d_{\mathrm{feature}}$ denote the Euclidean distances in geographic and feature space, respectively. The weighting coefficients 0.3 and 0.7 were determined through preliminary empirical evaluation; feature similarity receives a larger weight, whereas spatial proximity mainly serves to preserve geographical diversity. For each cluster with $N_{\mathrm{cluster}}>5$, the three samples with the smallest prediction errors are retained as representatives and the rest are eliminated, a threshold empirically set to balance intra-cluster coverage against redundancy.

The BALSO algorithm repeats this “forward selection–backward elimination” process iteratively. After each iteration, the model performance is evaluated on the current sample set $L_{\mathrm{current}}$. The optimization is considered to have converged when the root mean square error (RMSE) across all feature-space clusters falls below a preset threshold, $\theta_{\mathrm{RMSE}}=6.5\;t\cdot {\mathrm{ha}}^{-1}$, a value chosen through preliminary experiments to avoid excessive labeling costs for marginal gains.

### 2.6. DOA

In the algorithm’s initialization phase, the exploration phase iteration count is set to 60% of the total iteration count, and the grouping forgetting dimension array is defined as

$$k_{q}=\left[\left\lfloor \frac{\mathrm{Dim}}{5}\right\rfloor ,\left\lfloor \frac{\mathrm{Dim}}{4}\right\rfloor ,\left\lfloor \frac{\mathrm{Dim}}{3}\right\rfloor ,\left\lfloor \frac{\mathrm{Dim}}{2}\right\rfloor ,\left\lfloor \frac{\mathrm{Dim}}{1.5}\right\rfloor \right],$$

while the development phase forgetting dimension is

$$k_{r}=\left\lfloor \frac{\mathrm{Dim}}{3}\right\rfloor ,$$

and the strategy selection threshold is u=0.9. Here, ⌊·⌋ denotes the floor operator.

The dream population is initialized to generate *N* individuals, each representing a hyperparameter combination:

$$X=\left[\begin{array}{cccc} x_{1,1} & x_{1,2} & \cdots & x_{1,\mathrm{Dim}}\\ x_{2,1} & x_{2,2} & \cdots & x_{2,\mathrm{Dim}}\\ \vdots & \vdots & \ddots & \vdots \\ x_{N,1} & x_{N,2} & \cdots & x_{N,\mathrm{Dim}} \end{array}\right]$$

The position of each individual corresponds to a target function value:

$$F=[F\left(X_{1}\right)\;F\left(X_{2}\right)\;\cdots \;F\left(X_{N}\right)].$$

In the exploration phase (first 60% of iterations), the algorithm divides the population into five groups based on memory capacity, simulating individuals with different memory capacities. For each individual in a group, a memory strategy is used to reset its position to the optimal position within the group, and then $k_{q}\left[q\right]$ forgotten dimensions are randomly selected for updating. With a probability of 90%, a forgetting supplementation strategy is adopted to perform exploratory updates in the forgotten dimensions:

$$x_{t+1}\left(i,j\right)=\mathrm{current}\;\mathrm{position}\left[j\right]+r\;\left(\mathrm{ub}\left[j\right]-\mathrm{lb}\left[j\right]\right),$$

where r∼U(0,1) is a uniformly distributed random variable.

With a probability of 10%, the dream sharing strategy is adopted to obtain information from other individuals:

$$x_{t+1}\left(i,j\right)=\mathrm{current}\;\mathrm{position}\left[j\right]+r\;\left(x\left(m,j\right)-x\left(i,j\right)\right).$$

During the development phase (last 40% of iterations), all individuals learn from the global optimal position, randomly selecting $k_{r}$ forgotten dimensions for fine-tuning:

$$x_{t+1}\left(i,j\right)=\mathrm{g\_opt}\left[j\right]+\left(1-\frac{t}{T_{\max}}\right)g\left(\mathrm{ub}\left[j\right]-\mathrm{lb}\left[j\right]\right),$$

where g∼N(0,1) is a standard Gaussian random variable.

Boundary handling employs intelligent strategies: for low-dimensional problems (Dim≤15), traditional random reset methods are used; for high-dimensional problems (Dim>15), the dream sharing strategy is adopted for boundary handling to enhance global search capabilities.

Fitness evaluation is based on AGB estimation accuracy, measured by the $R^{2}$ value of five-fold cross-validation:

$$\mathrm{Fitness}\left[i\right]=5-\mathrm{fold}\;\mathrm{cross}-\mathrm{validation}\;R^{2}\left(\mathrm{model}\right).$$

DOA demonstrates significant advantages in parameter optimization through its unique dream memory mechanism. The grouping exploration strategy lets five memory groups explore different regions of the parameter space, so important regions are not easily omitted. The selective forgetting mechanism updates only 30–40% of the parameter dimensions, significantly reducing invalid evaluation times. The dream sharing strategy prevents population diversity from being lost through 10% global information exchange. The detailed procedure of DOA is summarized in Algorithm 1.

**Algorithm 1** Pseudocode for DOA Algorithm.
**Require:** $X_{\mathrm{sel}}$, $D_{\mathrm{opt}}$, *y*, hyperparameter search space**Ensure:** Optimal hyperparameter combination, optimal model  1:

$T_{d}\leftarrow 0.6\times T_{\max}$

  2:

$k_{q}\leftarrow \left[\left\lfloor \mathrm{Dim}/5\right\rfloor ,\left\lfloor \mathrm{Dim}/4\right\rfloor ,\ldots \right]$

  3:

$k_{r}\leftarrow \left\lfloor \mathrm{Dim}/3\right\rfloor$

  4:

u←0.9

  5:Population ← RandomInitialization(*N*, Dim)  6:**for** t=1 to $T_{\max}$ **do**  7:      **if** $t\leq T_{d}$ **then**  8:            Grouped_population ← MemoryGroup(Population, $k_{q}$)  9:            **for** q=1 to 5 **do**10:                   **for** *i* in Grouped_population[*q*] **do**11:                         CurrentPos ← best position within the group12:                         ForgottenDims ← RandomSelect(Dim, $k_{q}\left[q\right]$)13:                         **if** rand()<u **then**14:                               **for** *j* in ForgottenDims **do**15:                                     $x_{t+1}\left(i,j\right)\leftarrow \mathrm{CurrentPos}\left[j\right]+\mathrm{rand}\left(\right)\times \left(ub\left[j\right]-lb\left[j\right]\right)$16:                               **end for**17:                         **else**18:                               **for** *j* in ForgottenDims **do**19:                                     m← randomly select an individual index excluding *i*20:                                     $x_{t+1}\left(i,j\right)\leftarrow \mathrm{CurrentPos}\left[j\right]+\mathrm{rand}\left(\right)\times \left(x\left(m,j\right)-x\left(i,j\right)\right)$21:                               **end for**22:                         **end if**23:                   **end for**24:            **end for**25:      **else**26:            **for** i=1 to *N* **do**27:                   CurrentPos ← global optimum position28:                   ForgottenDims ← RandomSelect(Dim, $k_{r}$)29:                   **for** *j* in ForgottenDims **do**30:                         $x_{t+1}\left(i,j\right)\leftarrow g_{\mathrm{opt}}\left[j\right]+\left(1-t/T_{\max}\right)\times \mathrm{randn}\left(\right)\times \left(ub\left[j\right]-lb\left[j\right]\right)$31:                   **end for**32:            **end for**33:      **end if**34:      **for** i=1 to *N* **do**35:            **for** j=1 to Dim **do**36:                   **if** $x_{t+1}\left(i,j\right)<lb\left[j\right]$ or $x_{t+1}\left(i,j\right)>ub\left[j\right]$ **then**37:                         **if** Dim ≤15 **then**38:                               $x_{t+1}\left(i,j\right)\leftarrow lb\left[j\right]+\mathrm{rand}\left(\right)\times \left(ub\left[j\right]-lb\left[j\right]\right)$39:                         **else**40:                               m← randomly select an individual index excluding *i*41:                               $x_{t+1}\left(i,j\right)\leftarrow \mathrm{CurrentPos}\left[j\right]+\mathrm{rand}\left(\right)\times \left(x\left(m,j\right)-x\left(i,j\right)\right)$42:                         **end if**43:                   **end if**44:            **end for**45:      **end for**46:      **for** i=1 to *N* **do**47:            model ← train with current hyperparameters ($X_{\mathrm{selected}}$, $D_{\mathrm{optimized}}$)48:            Fitness[*i*] ← 5-fold cross-validation $R^{2}$(model)49:      **end for**50:      Update group optimal and global optimal (Population, Fitness)51:
**end for**
52:**return** global optimal hyperparameters, corresponding model


## 3. Experiments

A series of experiments were designed to validate whether the DFS framework is effective for forest AGB estimation. Ten-fold cross-validation was used as the main validation scheme, and it was repeated three times, for both the self-built and public datasets. All experiments were done under the same hardware and software environments, and the specific configurations and parameter settings are given in Table 2.

### 3.1. Model Construction and Evaluation

#### 3.1.1. Model Selection

To fully assess how well our proposed DFS framework performs and works with different machine learning algorithms, this study adopts three widely used regression models: Random Forest (RF), XGBoost, and LightGBM. This setup aims to test whether the DFS framework can consistently boost prediction accuracy and reduce computational overhead across different models.

#### 3.1.2. Model Evaluation Metrics

This study wants to comprehensively assess how different machine learning models perform in estimating forest AGB, so four regression metrics were adopted. They are the coefficient of determination ($R^{2}$), root mean square error (RMSE), relative root mean square error (rRMSE), and mean absolute error (MAE).

The coefficient of determination ($R^{2}$) quantifies the proportion of variance in the target variable explained by the model, and is calculated as:

$$R^{2}\left(y,\hat{y}\right)=1-\frac{\sum_{i=1}^{N}{\left(y_{i}-{\hat{y}}_{i}\right)}^{2}}{\sum_{i=1}^{N}{\left(y_{i}-\bar{y}\right)}^{2}}$$

RMSE is short for root mean square error, it is used to measure the average size of prediction errors, and its definition is given as follows:

$$\mathrm{RMSE}\left(y,\hat{y}\right)=\sqrt{\frac{1}{N}\sum_{i=1}^{N}{\left(y_{i}-{\hat{y}}_{i}\right)}^{2}}$$

The relative root mean square error (rRMSE) normalizes the RMSE by the mean of the observed values, providing a unitless measure of relative error:

$$\mathrm{rRMSE}=\frac{\mathrm{RMSE}}{\bar{y}}\times 100\%$$

The mean absolute error (MAE) represents the average absolute deviation between predicted and observed values, and is robust to outliers:

$$\mathrm{MAE}\left(y,\hat{y}\right)=\frac{1}{N}\sum_{i=1}^{N}\left|y_{i}-{\hat{y}}_{i}\right|$$

where $y_{i}$ and ${\hat{y}}_{i}$ are the observed and predicted AGB values for the *i*-th sample, respectively, $\bar{y}$ is the mean of the observed values, and *N* is the total number of samples. A higher $R^{2}$ (closer to 1) and lower values of RMSE, rRMSE, and MAE indicate better predictive accuracy and model robustness.

### 3.2. Module Effectiveness Experiments

In order to verify the independent contribution of each module in the DFS framework, we conducted module effectiveness experiments on DCAFS, BALSO and DOA on two self-built datasets in Hunan and Hubei.

#### 3.2.1. Performance of DCAFS for Feature Selection

To comprehensively evaluate the performance and innovation of the DCAFS algorithm, we designed comparative experiments from two perspectives. First, we compared DCAFS with three mainstream single-criterion feature selection methods: ReliefF, mutual information, and random forest feature importance. This comparison aimed to validate the superiority of integrating multiple importance criteria. Second, we compared DCAFS with the standard particle swarm optimization (PSO) feature selection method to demonstrate the effectiveness of its problem-aware mechanism and enhancements such as the diversity penalty term.

**Figure 3:**
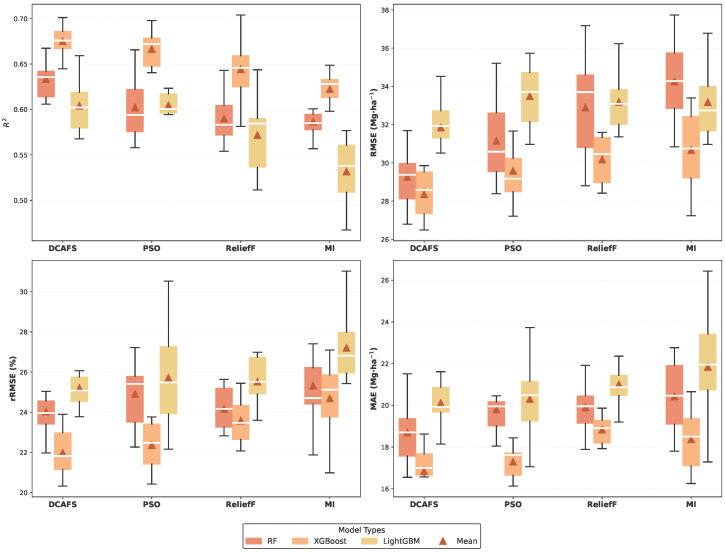
Box plot of RF, XGBoost and LightGBM model performance under different feature selection methods on the Hubei datase.

On the Hunan dataset, DCAFS selected 11 key features from 68 original features. As shown in Table 3, the feature subset selected by DCAFS achieved the best or near-best predictive performance across all three models. Taking XGBoost as an example, the average R^2^ of DCAFS was 0.68±0.02, outperforming the single-criterion methods and standard PSO. On the Hubei dataset, DCAFS selected 13 features and consistently demonstrated stable advantages, with balanced performance across models (Figure 3). The performance fluctuations observed with single-criterion methods across different models were substantially mitigated.

**Figure 4:**
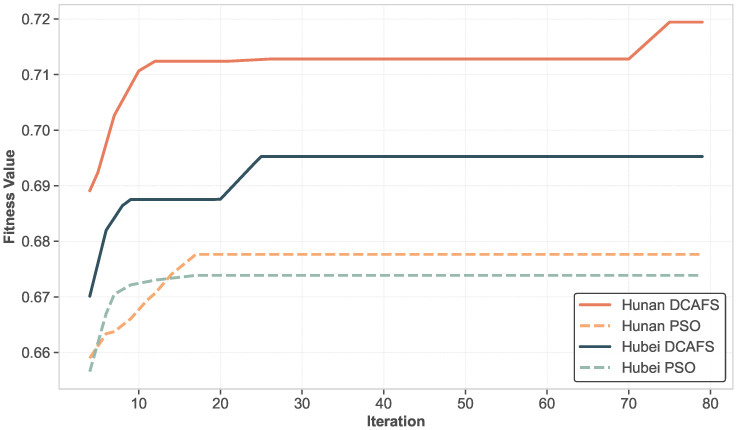
Comparison of convergence curves between DCAFS and standard PSO.

The convergence performance analysis (Figure 4) further reveals the efficiency of the DCAFS algorithm. DCAFS converged after approximately 40 iterations, about 33% faster than standard PSO (which required around 60 iterations), and achieved a better final fitness value. This improvement can be attributed to the integration of ReliefF and MI, which provided prior knowledge to guide the search direction, and the dynamic inertia weight mechanism, which finely tuned the search process and effectively prevented premature convergence.

**Figure 5:**
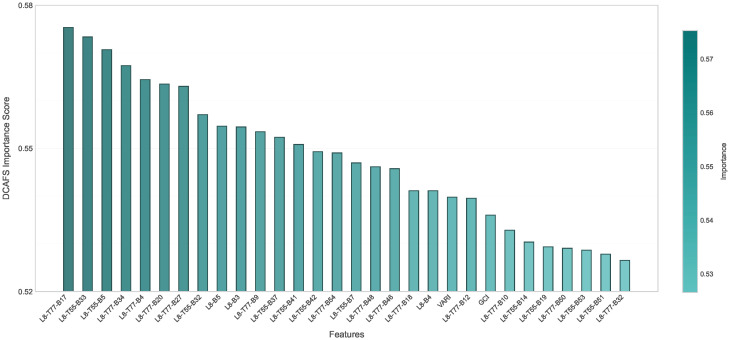
Bar chart of feature importance filtered by DCAFS on Hunan dataset.

**Figure 6:**
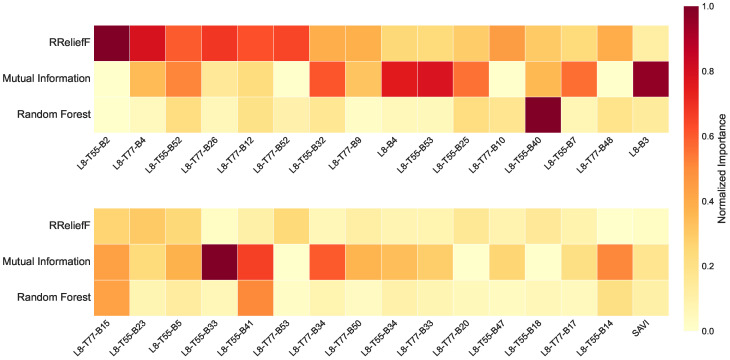
Heatmap of feature importance scores of DCAFS-selected features assessed by ReliefF, Mutual Information (MI), and Random Forest feature importance methods on the Hunan dataset.

The feature importance analysis was done, and Figure 5 shows the ranking of feature importance that DCAFS got on the Hunan dataset. The importance scores of the selected features are relatively uniform, indicating that DCAFS successfully avoids over-reliance on any single predictor and instead assembles a coalition of complementary biophysical indicators. Figure 6 further shows the consistency of the features that DCAFS selected across multiple evaluation criteria, and this was done by using a heatmap of importance scores from ReliefF, MI, and RF-FI, so it indicates that the selected features are quite interpretable and robust.

Comprehensive analysis demonstrates that the DCAFS algorithm not only overcomes the limitations of single-criterion feature selection by integrating multi-source criteria, but also substantially outperforms the original PSO framework in terms of search efficiency and solution quality through its problem-aware particle swarm improvement mechanism. It consistently achieves excellent performance across three markedly different models (RF, XGBoost, and LightGBM), demonstrating that the robustness of the selected feature subset is model-independent, thereby providing a highly reliable input for subsequent optimization modules.

#### 3.2.2. Optimization Effect of BALSO for Sampling

In order to verify the optimization ability of the BALSO algorithm under different sample distribution conditions, sample optimization experiments were carried out on two self-built datasets in Hunan and Hubei, and the optimization effect was evaluated by 10-fold cross-validation (3 repeats) on three representative models: random forest (RF), XGBoost and LightGBM.

**Figure 7:**
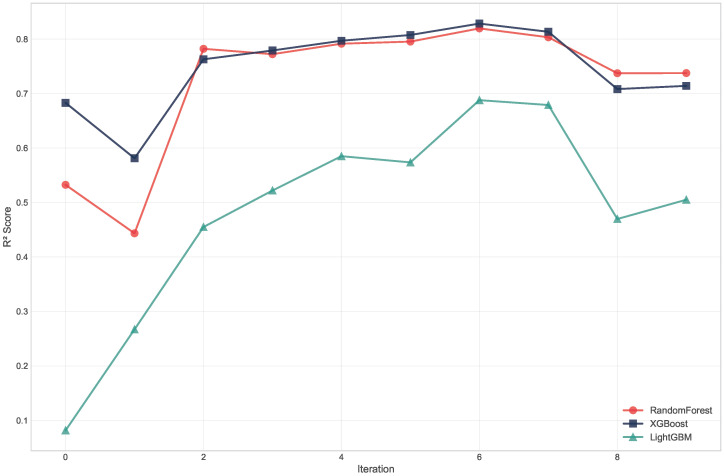
Comparison of convergence processes of the BALSO algorithm on the Hunan dataset.

The BALSO algorithm iteratively optimizes the training sample set through a bidirectional “forward selection–backward elimination” mechanism. On the Hunan dataset, the algorithm underwent 10 rounds of iterative optimization (Figure 7). During the initial iterations, the model performance fluctuated significantly as the sample composition changed substantially. After approximately 6 iterations, the R^2^ scores of RF and XGBoost gradually stabilized around 0.80–0.82, while LightGBM showed slower convergence, reaching a plateau near 0.69 after 6 iterations. This convergence pattern indicates that BALSO effectively identifies and retains high-information samples while eliminating redundant ones, though the optimization trajectory varies across model architectures.

**Figure 8:**
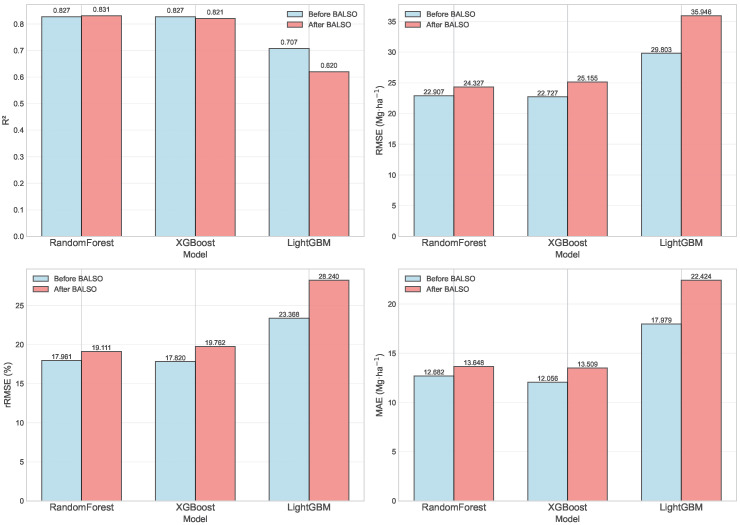
Comparison of model performance of the BALSO algorithm on the Hunan dataset.

To further investigate the interaction between BALSO and the other DFS modules, we examined the BALSO optimization process under the condition where features had already been selected by DCAFS and model parameters preliminarily tuned. Figure 8 presents the performance comparison before and after BALSO optimization in this setting. On the Hunan dataset, the final optimized sample set contained 332 samples. For RF, the R^2^ slightly changed from 0.827 to 0.831, while RMSE increased marginally from 22.91 to 24.33 Mg·ha^−1^, rRMSE from 17.96% to 19.11%, and MAE from 12.68 to 13.65 Mg·ha^−1^. For XGBoost, the R^2^ experienced a minor decrease from 0.827 to 0.821, with RMSE rising from 22.73 to 25.16 Mg·ha^−1^, rRMSE from 17.82% to 19.76%, and MAE from 12.06 to 13.51 Mg·ha^−1^. LightGBM exhibited the most pronounced changes: R^2^ decreased from 0.707 to 0.620, RMSE increased from 29.80 to 35.95 Mg·ha^−1^, rRMSE from 23.37% to 28.24%, and MAE from 17.98 to 22.42 Mg·ha^−1^.

These results indicate that when applied after DCAFS feature selection, BALSO achieves substantial sample compression (46.8% reduction) while maintaining relatively stable performance for RF and XGBoost. The slight performance fluctuations observed for RF and XGBoost, as well as the more noticeable degradation for LightGBM, can be attributed to the trade-off between sample size reduction and information preservation. The backward elimination phase removes spatially and feature-redundant samples, which may eliminate some marginally informative samples that are particularly beneficial for LightGBM’s leaf-wise growth strategy. Nevertheless, the optimized sample set exhibits more uniform distribution in the feature space, which helps alleviate the risk of model overfitting to local features and improves the overall representativeness of the training data. It should be noted that the absolute performance levels shown here differ from the ablation experiment results in Table 4 because the latter reflect the independent contribution of BALSO under default feature and parameter settings (Group A2), whereas Figure 8 illustrates BALSO’s behavior in the context of the full DFS pipeline.

The Hubei dataset reached convergence after 4 rounds of iterations, and the optimized sample set contained 172 samples. The multi-model performance verification confirmed the versatility of BALSO: the average R^2^ of the random forest model increased from 0.802 to 0.816, the RMSE decreased from 26.3 Mg·ha^−1^ to 25.4 Mg·ha^−1^; the average R^2^ of the XGBoost model increased from 0.808 to 0.822, the RMSE decreased from 25.8 Mg·ha^−1^ to 24.9 Mg·ha^−1^; and the average R^2^ of the LightGBM model increased from 0.805 to 0.819, RMSE decreased from 26.0 Mg·ha^−1^ to 25.2 Mg·ha^−1^. A total of 34 redundant samples were excluded at this stage, of which 72% were spatial-feature double redundant sample pairs. The optimized sample set is reduced by nearly half of the samples, and the performance of each model remains basically stable.

The two datasets both gave results that show BALSO can optimize sample distribution in different geographical environments and forest types, and it can also improve the information density and feature spatial representativeness of the sample set. The algorithm uses an intelligent two-way selection mechanism, so it can keep or even improve model performance while the cost of sample labeling is reduced, and this gives an effective technical solution for sample scarcity and uneven distribution in practical applications.

#### 3.2.3. Performance of DOA for Parameter Tuning

To evaluate the parameter optimization capability of the DOA algorithm, hyperparameter tuning experiments were conducted on the self-built Hunan and Hubei datasets. DOA was compared with six parameter optimization methods: Bayesian Optimization, Optuna-TPE, WOA [36], HHO [37], and SMA [38].

On the Hunan dataset, DOA demonstrated excellent performance in XGBoost hyperparameter optimization. In 10-fold cross-validation, DOA achieved an average R^2^ of 0.83±0.02, an average RMSE of 25.6±1.5 Mg·ha^−1^, an average rRMSE of 18.2±1.1%, and an average MAE of 19.5±1.2 Mg·ha^−1^. These results correspond to the complete DFS framework (Group C) in the ablation study (Table 4), confirming that DOA effectively identifies optimal hyperparameter combinations that maximize model performance.

**Figure 9:**
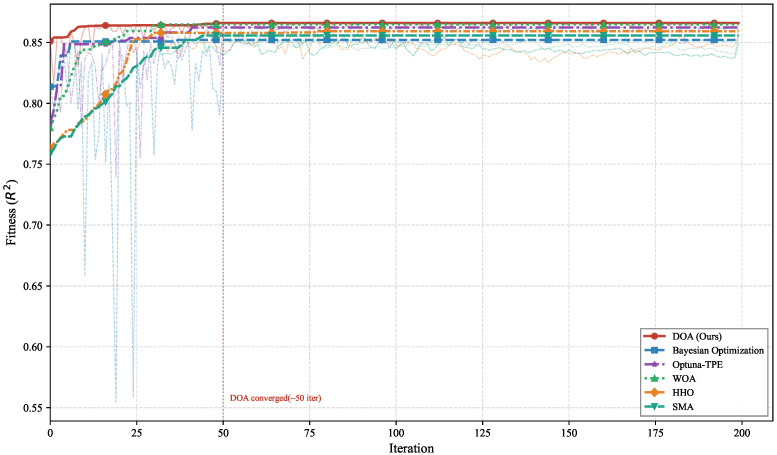
Comparison of convergence behavior and solution stability between DOA and other parameter optimization methods on the Hubei dataset. Thin translucent lines denote raw fitness trajectories at each iteration, while thick opaque lines represent the best-so-far envelope.

On the Hubei dataset, the validation results were generally consistent with the Hunan dataset. As illustrated in the convergence curves (Figure 9), DOA rapidly approached the optimal solution around the 50th iteration, achieving a final R^2^ score of approximately 0.865. In contrast, Bayesian Optimization and Optuna-TPE exhibited slower convergence with persistent fluctuations, while the modern metaheuristic algorithms WOA, HHO, and SMA required 80–120 iterations to approach comparable fitness levels and displayed larger-amplitude oscillations throughout the search process. This visualization confirms that DOA exhibits markedly smaller post-convergence oscillations compared to WOA and HHO. This demonstrates that the dream memory mechanism enables significantly faster convergence: the grouped exploration strategy allows five memory groups to explore different regions of the parameter space; the selective forgetting mechanism reduces invalid evaluations by updating only 30–40% of the parameter dimensions; and the dream sharing strategy prevents population diversity loss through 10% global information exchange.

Comprehensive experimental results from both datasets demonstrate that the DOA algorithm exhibits outstanding optimization performance compared to both surrogate-model-based methods and modern metaheuristic algorithms (WOA, HHO, SMA). Its dream memory mechanism demonstrates good generalization capability and stability across different forest datasets. DOA achieves final accuracy comparable to or better than Optuna-TPE, while requiring substantially fewer iterations to reach convergence than all competing metaheuristic methods.

### 3.3. Ablation Study

To evaluate the independent contributions and synergistic effects of the modules within the DFS framework, six ablation experiments were designed on the two self-constructed datasets from Hunan and Hubei (Table 4). All experiments were assessed using 10-fold cross-validation with three repetitions.

The experimental results demonstrate that the DFS framework exhibits stable optimization performance on both datasets. On the Hunan dataset, the complete DFS framework (Group C) achieved an average R^2^ of 0.83 ± 0.02, an average RMSE of 25.6 ± 1.5 Mg·ha^−1^, an average rRMSE of 18.2 ± 1.1%, and an average MAE of 19.5 ± 1.2 Mg·ha^−1^. A similar trend was observed for the Hubei dataset, where the complete framework achieved an average R^2^ of 0.86 ± 0.02, an average RMSE of 26.8 ± 1.6 Mg·ha^−1^, an average rRMSE of 18.6 ± 1.1%, and an average MAE of 20.2 ± 1.3 Mg·ha^−1^.

Independent analysis of the modules reveals that DCAFS substantially reduces feature dimensionality while preserving or even improving predictive accuracy. BALSO significantly compresses the training sample set and optimizes spatial–feature distribution, thereby alleviating redundancy. DOA further enhances estimation stability and accuracy when built upon the optimized feature and sample spaces. In the module combination experiment, the DCAFS+BALSO combination brought considerable savings in computational overhead on the two datasets, but exhibited a slight decrease in accuracy, underscoring the necessity of the parameter tuning module.

### 3.4. Comparative Experiments

To comprehensively evaluate the performance of the proposed DFS framework, this section presents comparative experiments on the Inner Mongolia public dataset (Wangyedian Forest Farm), encompassing baseline machine learning models, a representative deep learning method, and the state-of-the-art method by Xu et al. [20]. All experiments were conducted using identical 10-fold cross-validation with three repetitions and the four evaluation metrics introduced in Section 3.1.2.

As shown in Table 5, XGBoost without DFS optimization achieved an average $R^{2}$ of 0.77±0.03 and an RMSE of 21.5±1.5 Mg·ha^−1^. The DFS framework based on XGBoost improved the $R^{2}$ to 0.84±0.02 and reduced the RMSE to 18.1±1.0 Mg·ha^−1^, demonstrating the substantial accuracy gains from collaborative feature-sample-parameter optimization. We selected XGBoost as the representative ensemble model because it consistently outperformed RF and LightGBM in our module effectiveness experiments (Table 3); the DFS framework is model-agnostic and can be equivalently applied to other regression models.

To contextualize the DFS framework within the broader machine learning landscape, we further compared it with a Multi-Layer Perceptron (MLP), a representative deep learning approach for regression tasks. As shown in Table 5, the MLP model yielded an average $R^{2}$ of 0.71±0.04 and an RMSE of 24.1±1.8 Mg·ha^−1^. The MLP achieved lower prediction accuracy than the DFS-based XGBoost model, which is attributable to the limited sample size of the Inner Mongolia dataset. Deep learning models need large training sets. They also tend to overfit when samples are scarce [5]. This highlights the value of the DFS framework when field data are limited.

Finally, compared with the method proposed by Xu et al. [20], the DFS framework achieved better comprehensive prediction performance. While Xu et al.’s method focuses on stabilizing model output through secondary ensemble integration, the DFS framework addresses efficiency by jointly optimizing feature dimensionality, sample distribution, and parameter configuration.

### 3.5. Generalization Experiments

To evaluate the generalization ability of the proposed DFS framework, a cross-dataset validation experiment was designed. The Hunan and Hubei datasets have similar climates. They were used as mutual training and test sources to assess performance under similar conditions. The Inner Mongolia dataset comes from a different climate zone. It was used to test model robustness across regions.

It should be noted that the Inner Mongolia dataset comprises only 81 sample plots. If used as a training source, the sample size remaining after BALSO optimization would be too small to support reliable training of ensemble models, leading to a high risk of overfitting. Therefore, Inner Mongolia was designated as an independent test benchmark only, while Hunan and Hubei served as mutual training sources and test targets.

The cross-dataset generalisation results are shown in Table 6, and from these results it can be seen that model transferability is closely related to climate zones. When the source and target datasets are located within the same subtropical climate zone, the performance decline is relatively small, so the DFS framework can maintain a comparatively strong generalisation ability in similar ecoregions. However, when the transfer crosses different climate zones, a more obvious performance drop is observed.

## 4. Discussion

The DFS framework achieves synergistic optimization of feature dimensionality, sample distribution, and model hyperparameters for forest AGB estimation, suggesting that joint optimization of inputs and configuration is more cost-effective than increasing model complexity under resource constraints.

The DCAFS module addresses high-dimensional feature redundancy. Conventional filter methods ignore redundancy among features [19]. Standard wrapper methods such as PSO often converge prematurely [20]. Recent variants, including problem-aware PSO [29] and optimized LASSO [30], remain under-explored in heterogeneous forests [22]. DCAFS converts ReliefF and MI scores into PSO search-guidance signals, and it penalizes collinearity through a diversity reward. Table 3 shows that DCAFS achieved the best or near-best performance across all three models with fewer features. Figure 4 confirms that this mechanism converges faster.

Active learning has been increasingly applied to sample optimization in remote sensing AGB estimation [26,32,39]. However, existing strategies predominantly rely on forward selection, iteratively adding informative samples while neglecting redundancy accumulation within the labeled set [31]. This unidirectional mechanism introduces distribution bias when ground surveys are constrained by terrain and resources. BALSO bridges this gap through a bidirectional process: the forward phase integrates uncertainty and representativeness to prioritize information-rich samples, while the backward phase employs density peak clustering based on a comprehensive spatial-feature distance metric to eliminate redundant plots. For instance, on the Hunan dataset, this strategy removed a substantial proportion of redundant samples and maintained stable predictive performance (Figure 8).

Hyperparameter optimization in AGB estimation has traditionally relied on random search, or Bayesian optimization, which struggle to balance efficiency and convergence stability in high-dimensional, non-continuous spaces [27]. Cognition-inspired algorithms [34] offer alternatives but remain sensitive to parameter settings and data heterogeneity. DOA addresses these limitations through a dream-inspired memory mechanism. On the Hubei dataset, DOA approached convergence by the 30th iteration and achieved an $R^{2}$ of approximately 0.865 (Figure 9). The ablation study (Table 4) further indicates that DOA provides a necessary corrective gain when combined with DCAFS and BALSO, compensating for information loss induced by aggressive feature and sample compression.

When evaluated against existing methodologies, the DFS framework demonstrates clear advantages. Unlike Xu et al. [20], which stabilizes outputs through secondary ensemble integration, DFS improves efficiency from the data source by jointly optimizing features, samples, and parameters, yielding superior accuracy on the Inner Mongolia dataset (Table 5). Moreover, the underperformance of the Multi-Layer Perceptron (MLP) under limited-sample conditions confirms the data-hungry nature of deep learning architectures [5], suggesting that intelligently optimizing traditional ensemble models offers a more practical alternative in resource-constrained scenarios.

Despite these promising results, this study has several limitations that warrant critical examination.

(1) Input data quality. Landsat 8 SR products underwent USGS radiometric calibration and atmospheric correction. However, selecting scenes with cloud cover below 10% cannot fully eliminate residual atmospheric noise or cloud contamination. Temporal mismatch between the self-constructed and Inner Mongolia datasets may also introduce phenological inconsistencies.
(2) Satellite image seasonality. The current framework relies on single-date imagery, which does not capture intra-annual phenological dynamics. In subtropical forests, seasonal variation in evergreen and deciduous proportions alters spectral signatures and vegetation index stability, thereby modulating the feature–AGB relationship across acquisition dates. Future work should integrate multi-seasonal composites or phenological metrics from dense time-series to improve temporal robustness.
(3) Forest structural heterogeneity and interregional transfer limitations. The study areas span markedly different forest structures; differences in canopy height, stand density and vertical complexity alter the penetration and scattering of remote sensing signals, modifying the feature-AGB relationship. The cross-dataset results (Table 6) corroborate this, showing a clear performance decline when transferring from subtropical to temperate zones. Furthermore, the applicability to other ecosystem types remains untested, as species composition and background reflectance differ fundamentally across biomes, potentially inducing covariate shift [4]. Biome-specific calibration of feature-AGB relationships is often necessary [17,40], so the operational validity of DFS at global scales has not yet been demonstrated.
(4) Spatial autocorrelation and over-adaptation risks. Standard cross-validation may inflate performance metrics for spatial data, as adjacent plots share similar environmental conditions. We acknowledge this limitation and recommend spatial block cross-validation or leave-location-out validation for future studies. Meanwhile, although DCAFS was consistently applied across datasets without modification, the possibility of over-adaptation to specific feature spaces cannot be fully excluded, particularly given forest ecosystem heterogeneity across biomes [34].

## 5. Conclusions

This study proposes the DFS framework for regional-scale forest AGB estimation. It jointly addresses feature redundancy, uneven sample distribution, and inefficient hyperparameter optimization. Three core modules are integrated: DCAFS, BALSO, and DOA. These modules improve estimation accuracy while reducing computational and data costs.

Experimental validation on two subtropical forest datasets demonstrated that the DFS framework achieves robust predictive performance, with $R^{2}$ values of 0.83 and 0.86 and RMSE values of 25.6 and 26.8 Mg·ha^−1^, respectively. The framework also maintained satisfactory accuracy on an independent public dataset from Inner Mongolia, indicating its potential for broader application. Through the collaborative optimization of features, samples, and parameters, the framework substantially reduces input dimensionality and labeling burden without sacrificing predictive accuracy.

Several limitations should be acknowledged. First, the full operational validity of the DFS framework has not yet been demonstrated, because the current validation is restricted to three study areas spanning subtropical and mid-temperate climate zones; global-scale testing and independent field data from additional biomes are still lacking. Second, the impacts of input data quality, satellite image seasonality, and complex forest structural heterogeneity on model stability have not been thoroughly quantified. Third, the DOA algorithm entails higher computational cost than lightweight heuristic methods, and its sensitivity to built-in parameters warrants further investigation across more diverse datasets.

Despite these limitations, the proposed framework offers a feasible and efficient approach for regional forest AGB estimation and carbon monitoring, particularly in resource-constrained settings where in-situ data are limited. Future work will focus on extending the framework to diverse ecological zones, integrating deep learning-based feature extraction, and developing interactive decision-support tools for operational forest carbon assessment. Also adopt spatial block cross-validation for unbiased error estimation and assimilate multi-seasonal imagery to account for phenological variability.

## Figures and Tables

**Table 1:** List of vegetation indices calculated in this study.

Vegetation Index Name	Abbreviation	Calculation Formula
Ratio Vegetation Index	RVI	NIR/Red
Difference Vegetation Index	DVI	NIR−Red
Normalized Difference Vegetation Index	NDVI	(NIR−Red)/(NIR+Red)
Modified Ratio Vegetation Index/Simple Ratio Index	mRVI/MSR	(NIR/Red−1)/(NIR/Red+1)
Soil-Adjusted Vegetation Index	SAVI	(1+L)×(NIR−Red)/(NIR+Red+L),L=0.5
Modified Soil-Adjusted Vegetation Index	MSAVI	$2\times \mathrm{NIR}+1-\sqrt{{\left(2\times \mathrm{NIR}+1\right)}^{2}-8\times {\left(\mathrm{NIR}-\mathrm{Red}\right)}^{2}}$
Atmospherically Resistant Vegetation Index	ARVI	(NIR−(2×Red−Blue))/(NIR+(2×Red−Blue))
Enhanced Vegetation Index	EVI	2.5×(NIR−Red)/(NIR+6×Red−7.5×Blue+1)
Transformed Vegetation Index	TVI	(NIR−Red)/(NIR+Red)+0.5
Red-Green Vegetation Index	RGVI	Red/Green

**Table 2:** Experimental environment and parameter settings.

Parameters	Setting
Hardware	AMD Ryzen 9 8945HX (Advanced Micro Devices, Santa Clara, CA, USA)
	NVIDIA RTX 5060 Laptop GPU (NVIDIA Corporation, Santa Clara, CA, USA)
	32 GB RAM
Software	Python 3.13
Optimization alg. params	PSO: pop. size = 50, iter. = 100
	DOA: pop. size = 30, iter. = 200

**Table 3:** Performance comparison of feature selection methods on Hunan dataset (Mean ± SD of 10-fold cross-validation).

Algorithm	Feature Num	Model	$R^{2}$	RMSE (Mg·ha^−1^)	rRMSE (%)	MAE (Mg·ha^−1^)
DCAFS	11	RF	0.62 ± 0.03	31.0 ± 2.2	24.4 ± 1.7	19.1 ± 1.3
		XGBoost	0.68 ± 0.02	28.0 ± 1.8	22.0 ± 1.4	16.9 ± 1.1
		LightGBM	0.60 ± 0.04	32.2 ± 2.4	25.3 ± 1.9	19.9 ± 1.5
PSO	30	RF	0.60 ± 0.03	31.8 ± 2.2	25.0 ± 1.7	19.6 ± 1.3
		XGBoost	0.66 ± 0.02	28.9 ± 1.9	22.7 ± 1.5	17.5 ± 1.1
		LightGBM	0.58 ± 0.04	32.9 ± 2.5	25.9 ± 2.0	20.3 ± 1.6
ReliefF	40	RF	0.59 ± 0.03	32.3 ± 2.3	25.4 ± 1.8	19.9 ± 1.4
		XGBoost	0.64 ± 0.03	29.8 ± 2.0	23.4 ± 1.6	18.3 ± 1.2
		LightGBM	0.56 ± 0.04	33.7 ± 2.6	26.5 ± 2.0	20.9 ± 1.7
MI	27	RF	0.58 ± 0.03	32.7 ± 2.3	25.7 ± 1.8	20.2 ± 1.4
		XGBoost	0.63 ± 0.03	30.3 ± 2.0	23.8 ± 1.6	18.7 ± 1.2
		LightGBM	0.55 ± 0.04	34.2 ± 2.7	26.9 ± 2.1	21.2 ± 1.7
RF_FI	5	RF	0.57 ± 0.04	33.1 ± 2.5	26.0 ± 2.0	20.5 ± 1.6
		XGBoost	0.62 ± 0.03	30.8 ± 2.1	24.2 ± 1.7	19.0 ± 1.3
		LightGBM	0.54 ± 0.04	34.6 ± 2.8	27.2 ± 2.2	21.5 ± 1.8
All_Features	68	RF	0.59 ± 0.03	32.2 ± 2.3	25.3 ± 1.8	19.8 ± 1.4
		XGBoost	0.65 ± 0.02	29.2 ± 1.9	22.9 ± 1.5	17.8 ± 1.1
		LightGBM	0.57 ± 0.04	33.5 ± 2.6	26.3 ± 2.0	20.7 ± 1.7

**Table 4:** Ablation experiment results of DFS framework (Mean ± SD of 10-fold cross-validation).

Dataset	Group	DCAFS	BALSO	DOA	$R^{2}$	RMSE (Mg·ha^−1^)	rRMSE (%)	MAE (Mg·ha^−1^)	$N_{f}$	$N_{s}$
Hunan	Baseline	×	×	×	0.65 ± 0.02	29.4 ± 1.8	22.8 ± 1.6	18.1 ± 1.2	68	625
Hunan	A1	✔	×	×	0.68 ± 0.02	28.0 ± 1.7	21.9 ± 1.5	17.0 ± 1.2	11	625
Hunan	A2	×	✔	×	0.75 ± 0.03	31.2 ± 2.1	22.5 ± 1.8	21.6 ± 1.6	68	332
Hunan	A3	×	×	✔	0.66 ± 0.01	28.8 ± 1.8	22.3 ± 1.5	18.2 ± 1.3	68	625
Hunan	B	✔	✔	×	0.78 ± 0.02	29.0 ± 2.0	20.8 ± 1.5	20.1 ± 1.5	11	332
Hunan	C (Complete)	✔	✔	✔	0.83 ± 0.02	25.6 ± 1.5	18.2 ± 1.1	19.5 ± 1.2	11	332
Hubei	Baseline	×	×	×	0.64 ± 0.04	30.5 ± 1.9	21.1 ± 1.5	23.2 ± 1.7	68	312
Hubei	A1	✔	×	×	0.66 ± 0.02	29.2 ± 1.8	20.2 ± 1.4	22.2 ± 1.6	13	312
Hubei	A2	×	✔	×	0.60 ± 0.03	32.4 ± 2.2	22.8 ± 1.8	24.6 ± 1.9	68	172
Hubei	A3	×	×	✔	0.65 ± 0.02	29.9 ± 1.9	20.6 ± 1.4	22.4 ± 1.6	68	312
Hubei	B	✔	✔	×	0.64 ± 0.03	30.2 ± 2.1	21.0 ± 1.6	23.0 ± 1.8	13	172
Hubei	C (Complete)	✔	✔	✔	0.86 ± 0.02	26.8 ± 1.6	18.6 ± 1.1	20.2 ± 1.3	13	172

Note: $N_{f}$: number of selected features; $N_{s}$: number of optimized samples; ✔: module enabled; ×: module disabled.

**Table 5:** Performance comparison of different methods on the Inner Mongolia public dataset.

Method	$R^{2}$	RMSE (Mg·ha^−1^)	rRMSE (%)	MAE (Mg·ha^−1^)
Xu et al. [20]	0.81±0.03	19.5±1.4	18.9±1.3	15.3±1.1
XGBoost	0.77±0.03	21.5±1.5	20.8±1.4	16.8±1.2
MLP	0.71±0.04	24.1±1.8	23.4±1.7	19.0±1.5
DFS (ours)	0.84±0.02	18.1±1.0	17.3±0.8	14.0±0.9

**Table 6:** Cross-dataset generalization results.

Training Set	Test Set	$R^{2}$	RMSE (Mg·ha^−1^)	rRMSE (%)	MAE (Mg·ha^−1^)
Hunan	Hunan	0.83 ± 0.02	25.6 ± 1.5	18.2 ± 1.1	19.5 ± 1.2
Hunan	Hubei	0.82 ± 0.03	24.8 ± 1.6	17.2 ± 1.2	18.5 ± 1.1
Hunan	Inner Mongolia	0.75 ± 0.02	18.7 ± 1.1	19.7 ± 1.2	14.8 ± 0.9
Hubei	Hunan	0.82 ± 0.02	25.1 ± 1.4	17.8 ± 1.0	18.8 ± 1.1
Hubei	Hubei	0.86 ± 0.02	26.8 ± 1.6	18.6 ± 1.1	20.2 ± 1.3
Hubei	Inner Mongolia	0.78 ± 0.04	18.3 ± 1.1	19.3 ± 1.2	15.0 ± 0.9

## Data Availability

Landsat 8 and Sentinel-1/2 data are publicly available from Google Earth Engine and the Copernicus Open Access Hub. The processed feature matrices for Hunan and Hubei, including plot-level AGB and remote sensing features, are available on Zenodo (DOI: 10.5281/zenodo.21698424). Precise geographic coordinates have been removed per national forest resource confidentiality regulations. Original field inventory data remain subject to institutional restrictions; academic access may be requested via the corresponding author.
